# Correlation Analysis Between Time Awareness and Morningness-Eveningness Preference

**DOI:** 10.5334/jcr.225

**Published:** 2023-10-11

**Authors:** Reimi Sogawa, Fuminori Ono, Masahiko Terao, Shunta Nagano, Junko Kawabe, Koichi Node, Makoto Akashi

**Affiliations:** 1 Department of Clinical Genetics and Genomic Medicine, Okayama University Hospital, 2-5-1 Shikata-cho, Kitaku, Okayama, Japan; 2 Faculty of Education, Yamaguchi University, 1677-1 Yoshida, Yamaguchi, Yamaguchi, Japan; 3 The Research Institute for Time Studies, Yamaguchi University, 1677-1 Yoshida, Yamaguchi, Yamaguchi, Japan; 4 Department of Cardiovascular Medicine, Saga University, 5-1-1 Nabeshima, Saga, Saga, Japan

**Keywords:** time awareness, morningness-eveningness preference

## Abstract

The circadian clock is adjusted by light inputs via the retinohypothalamic tract. Because environmental light is controllable for modern humans at the individual’s preference although under social schedules, individual differences in time-related psychology and behavior may be associated with morningness-eveningness preference (M-E preference). To examine this hypothesis, we used the Time Management Scale and Time Anxiety Scale to quantify time-related psychology and behavior. These scales aim to evaluate “awareness of effective time management and utilization” and “anxiety about uncontrollable time schedule and unexpected time-related outcome”, respectively. According to our correlation analysis using mid-sleep time as a marker for M-E preference, we obtained results supporting our hypothesis in the correlation between the M-E preference values and the Time Management Scale scores, with larger “time estimation” and “taking each moment as it comes” scores associated with more morningness and eveningness, respectively. Considering that modern humans likely become night owls under artificial light conditions, it appears plausible that lower awareness of time management leads to more eveningness.

## Introduction

Almost all organisms on earth exhibit circadian rhythms in physiology and behavior, which are driven by the circadian clock [1,2,3]. This clock activates gene expression at appropriate times of the day, allowing organisms to adapt to the earth’s rotation. The hypothalamic suprachiasmatic nucleus, the central clock, orchestrates peripheral clocks, which are ubiquitous throughout the body and consist of cell-autonomous negative feedback loops of clock gene transcription [4,5]. These feedback loops drive circadian rhythms in physiology and behavior [6,7]. Importantly, the circadian clock is reset or adjusted mainly in response to environmental light.

It has been reported that human circadian rhythms show an individual difference in morningness-eveningness preference (M-E preference) [8,9]. Two major factors likely cause this preference. First, circadian period length of the cell-autonomous circadian oscillator driven by clock genes may contribute to the M-E preference. More specifically, several previous studies have suggested that a longer circadian period in the cell-autonomous circadian clock correlates with more eveningness [10,11,12]. Second, individual differences in circadian input pathways may affect the M-E preference. More specifically, the human circadian clock is adjusted mainly by light inputs via the retinohypothalamic tract [13], and some previous studies have suggested that individual difference in light environment and light sensitivity therefore influences the M-E preference [14,15,16].

It has been reported that M-E preference in humans correlates with psychological and emotional factors, although the causal link remains unclear. For example, more morningness is likely associated with higher conscientiousness, while more evening types tend to be more neurotic [17]. In addition, there is a significant relationship between eveningness and low self-control [18,19]. Related to this relationship, risk-taking behavior is likely associated with M-E preference, with evening types reporting higher levels of risk-taking [20,21,22]. Furthermore, several studies have indicated that emotional variables correlate with M-E preference [23,24,25,26]. In the present study, we focused on the fact that the light environment surrounding modern humans is predominantly affected by artificial light derived from room illumination and light-emitting devices, and that when and how these are used varies among individuals, dependent on individual differences in awareness and values of clock and social time. Given this background, we aimed to investigate the possibility that time-related psychology and behavior have some influence on the M-E preference.

The light environment surrounding modern humans is much more dependent on social schedule than sunrise and sunset in nature. Specifically, modern humans likely manage their daily schedule based on business and school time, in accordance with which bedtime (light-off) and wake-up time (light-on) are determined although at their own discretion. Individual difference in psychology and behavior related to time management may therefore be associated with different light environments among individuals, which should subsequently lead to individual differences in M-E preference. Here, we tested this hypothesis using the Time Management Scale and Time Anxiety Scale in order to quantify time-related psychology and behavior. Simply, these scales are used to evaluate “awareness of effective time management and utilization” and “anxiety about uncontrollable time schedule and unexpected time-related outcome”, respectively [27,28]. M-E preference was quantified using the Munich Chronotype Questionnaire (MCTQ) [29]. The possibility that psychology and behavior related to time management affect the M-E preference was examined by performing a correlation analysis between scores of the Time Management Scale or Time Anxiety Scale and values of the MCTQ.

## Results

The results obtained from 362 Japanese subjects using the Japanese version of the Munich Chronotype Questionnaire (MCTQ) show that the midpoint of sleep on free days (MSF), midpoint of sleep on workdays (MSW) and relative social jetlag (SJLrel) are 3:33 ± 1:06 (s.d.), 4:22 ± 1:36 (s.d.) and 0:48 ± 0:58 (s.d.), respectively (Table 1). These values are similar to those reported in a previous study in 450 Japanese subjects, in which the average MSW and MSF were 3.64 h and 4.66 h, respectively [30], suggesting that our data collection was successful and reliable.

**Table 1:** Demographic characteristics, M-E preference and time awareness of subjects.


Subject, no.	362

Sex, male/female, no.	201/160

Age, y. (s.d.)	29.6 (14.4)

Munich Chronotype Questionnaire (hh:mm)	

Midpoint of sleep on workdays, MSW (s.d.)	3:33 (1:06)

Midpoint of sleep on free days, MSF (s.d.)	4:22 (1:36)

Relative social jetlag, SJLrel (s.d.)	0:48 (0:58)

Time Management Scale	

Time estimation (s.d.)	20.6 (4.2)

Time utilization (s.d.)	16.0 (3.6)

Taking each moment as it comes (s.d.)	13.2 (3.2)

Time Anxiety Scale	

Time anxiety (s.d.)	23.0 (6.2)

Time irritation (s.d.)	21.8 (6.7)


**Figure 1:**
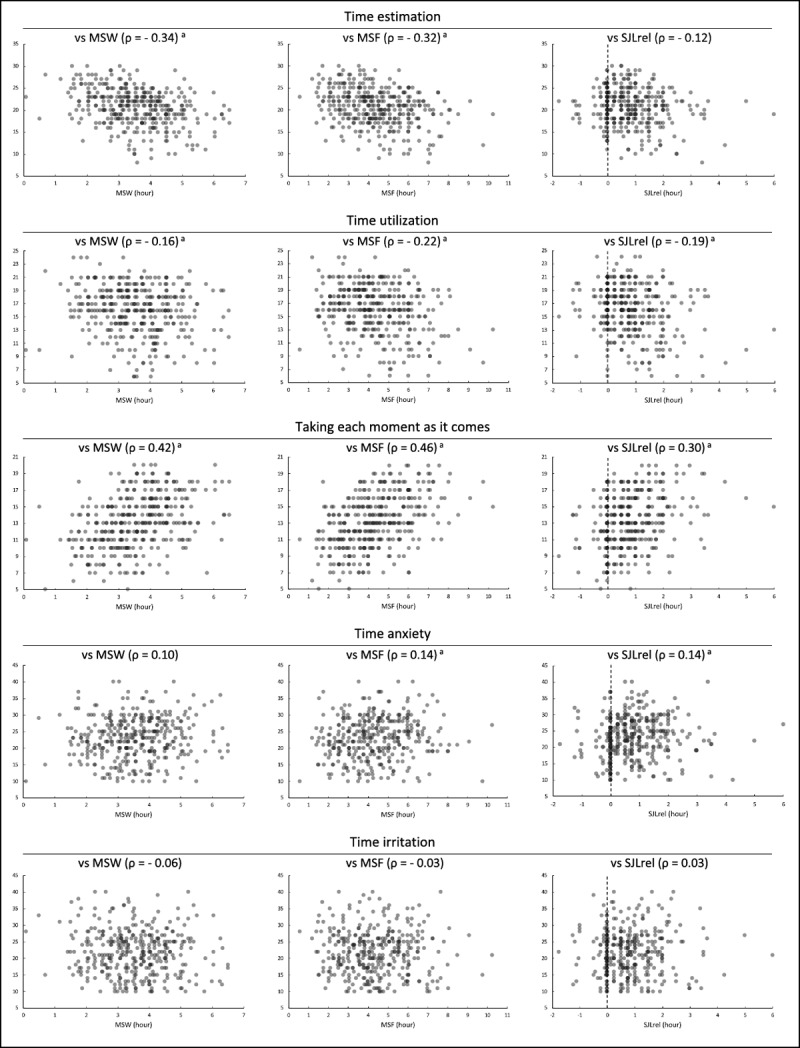
**Correlation analysis between M-E preference and time awareness**. To examine the correlation of MSW, MSF or SJLrel with “time estimation”, “time utilization”, “taking each moment as it comes”, “time anxiety” or “time irritation”, a rank correlation analysis was performed based on two-dimensional scatter plots. Each dot indicates data from one subject. ρ values represent correlation coefficients. “a” represents statistical significance (P < 0.01).

A rank correlation analysis was performed between M-E preference (values of the MCTQ) and time awareness (sub-scale scores of the Time Management Scale and Time Anxiety Scale) (Figure 1). Overall, the results showed small or moderate correlations between MCTQ value and Time Management Scale score, but no correlations (ρ < 0.2) between MCTQ value and Time Anxiety Scale score. Most sub-scale scores of the Time Management Scale correlated with MSW, MSF and SJLrel in a statistically significant manner. In particular, the findings showed the presence of a moderate positive correlation in four combinations between “time estimation” or “taking each moment as it comes” and MSW or MSF. Among the combinations, the two-dimensional scatter plot shows a clear positive correlation between “taking each moment as it comes” and MSW or MSF; the ρ values in these two combinations are 0.42 and 0.46, respectively. These results suggest a potential causal link between M-E preference and time management.

A rank correlation analysis was reperformed to examine differences in sex or age. While no obvious sex-dependent difference was detected (Figure S1), subjects aged ≤22 years likely showed lower correlations between “time estimation” or “taking each moment as it comes” and M-E preference than subjects aged ≥23 years (Figure S2). Given that most of the younger and older subjects were students and workers respectively, the difference in some social pressure(s) surrounding subjects may have contributed to these differences in correlation between the two groups. However, conclusive results require a larger sample size.

## Discussion

Circadian rhythms in behavior and physiology are driven by cell-autonomous negative feedback loops in clock gene expression. Circadian characteristics in the molecular oscillator should therefore have a major influence on M-E preference. Indeed, many studies using clock gene deficient and mutant mice have reported that cell-autonomous circadian characteristics reflect those at the whole body level [31,32,33]. In particular, circadian period length among these characteristics are well associated with M-E preference. For example, *clock* mutant mice with a long circadian period show circadian locomotor activity like delayed sleep phase disorder under normal light and dark conditions [34]. Also in humans, clock gene mutations and polymorphisms are associated with M-E preference [35]; an outstanding example of this is a *Per2* mutation causing familial advanced sleep phase disorder [36].

Environmental light surrounding humans also contributes to individual differences in M-E preference, because the mammalian circadian clock is adjusted mainly by light input via the retinohypothalamic tract. Importantly, the average circadian phase of melatonin secretion (closely related to M-E preference) in modern humans under artificial light conditions is significantly delayed in comparison with that under natural light conditions [37]. Environmental light is now controllable by modern humans, dependent on the individual’s preferences in the use of room illumination and light-emitting devices. Individual psychology and values related to this preference therefore likely have some influence on M-E preference. Given that most modern humans need to manage daily time based on a social schedule at their own discretion, and that light-related environmental cues are strongly associated with this schedule, we hypothesized that individual differences in psychology and behavior related to time management contribute to M-E preference.

Possible psychology and behavior related to daily time management are as follows. On workday evenings, some people stay up late studying or relaxing against homeostatic sleep pressure and circadian-driven sleepiness, while others go to bed before becoming sleepy in preparation for business or school the next day. On workday mornings, some get up early to utilize their morning time effectively, while others get up just in time for business or school. In addition, in the case of holidays, some get up late to reduce the sleep debt accumulated during the workdays, while others are active in a planned way from early morning. Together, levels of awareness that people bring in estimating time in advance and utilizing it effectively, or/and levels of anxiety in people who feel time-starved and pressed for time may contribute to decision making on daily time management. We therefore hypothesized that this awareness and anxiety may cause individual differences in M-E preference in a non-negligible manner. In this study, we verified this hypothesis by quantifying this awareness and anxiety using the Time Management Scale and Time Anxiety Scale.

According to our correlation analysis using mid-sleep time as a marker for M-E preference, we obtained results supporting our hypothesis in the correlation between M-E preference values and Time Management Scale scores. More specifically, a reasonable correlation was detected as below: larger “time estimation” and “taking each moment as it comes” scores were associated with more morningness and eveningness, respectively. In particular, the latter correlation was higher on free days, therefore likely resulting in a positive correlation between SJLrel and “taking each moment as it comes”. These results may be considered acceptable without inconsistency, as follows. Light input for modern humans is too weak in the morning and too strong at night in comparison with natural conditions, as a result of our spending many hours of the day under indoor lighting. Considering human phase response curves for circadian light input [38,39,40], this strongly suggests that modern humans likely become night owls under artificial light conditions. It therefore appears plausible that lower awareness of daily time management leads to more eveningness. Unexpectedly, we saw no correlation between M-E preference value and the Time Anxiety Scale score. This result was surprising, because we considered that higher anxiety about an uncontrollable time schedule and unexpected time-related outcome would enhance awareness of daily time management.

As a major limitation of this study, the present correlation analysis cannot exclude a reverse causal link, namely that M-E preference affects awareness of time management. Future studies using many more subjects may allow correlation analysis considering differences in age and sex, and provide further interesting information.

## Methods

### Ethics statement

All procedures in this study were approved by the institutional review boards of Yamaguchi University, with all subjects providing written informed consent.

### Subjects

A total of 362 Japanese subjects (201 males, 160 females and one unknown) with an average age of 29.6 ± 14.4 (SD) years were recruited for this study (Table 1). To quantify morningness-eveningness preference (M-E preference), all subjects completed the Japanese version of the Munich Chronotype Questionnaire (MCTQ). This is a useful tool for obtaining well-defined sleep parameters, such as the midpoint of sleep on free days (MSF), midpoint of sleep on workdays (MSW) and relative social jetlag (SJLrel) [41]. In addition, to quantify awareness of time, they also completed the Japanese version of the Time Management Scale and Time Anxiety Scale, which measures anxiety about uncontrollable time schedules and unexpected time-related outcomes [27,28]. These scales consist of three sub-scales (“time estimation”, “time utilization” and “taking each moment as it comes”) and two sub-scales (“time anxiety” and “time irritation”), respectively. English versions are shown in Supplemental Information (note that these versions remain unverified). For each question item, «not applicable», «somewhat applicable», «somewhat applicable» or «applicable» was scored as 1, 2, 3 or 4, respectively. In the Time Management Scale, “time estimation”, “time utilization” or “taking each moment as it comes” was calculated as the total score of answers to questions 1 to 8, questions 9 to 14 or questions 15 to 19, respectively (note that the answer to question 19 was scored in reverse). For the Time Anxiety Scale, “time anxiety” or “time irritation” was calculated as the total score of answers to questions 1 to 10 or questions 11 to 20, respectively. The internal consistency of the Time Management Scale and Time Anxiety Scale in Japanese populations was evaluated based on Cronbach›s coefficient alpha and split-half correlations, respectively, in the original papers [27,28]. In the former scale, the values of Cronbach›s coefficient alpha were “time estimation”, 0.83; “time utilization”, 0.80; and “taking each moment as it comes”, 0.77. In the latter scale, the split-half coefficient values were “time anxiety”, 0.82, and “time irritation”, 0.81.

### Statistical analysis

The strength of links of three values of the MCTQ (MSW, MSF and SJLrel) with five sub-scale scores of the Time Management Scale and Time Anxiety Scale (“time estimation”, “time utilization”, “taking each moment as it comes”, “ time anxiety” and “time irritation”) was examined using Spearman’s rank correlation. Spearman’s rank correlation coefficient, ρ, was used to evaluate the strength of links between data. A p-value less than 0.01 was considered statistically significant.

## Data Accessibility Statement

All data are included in this manuscript and its supplementary information files.

## Additional Files

The additional files for this article can be found as follows:

10.5334/jcr.225.s1 Figure S1. **Sex-dependent difference in the correlation between M-E preference and time awareness.** The rank correlation analysis in Figure 1 was reperformed by gender. Each blue or red dot indicates data from a male (m) or female (f) subject. ρ values represent correlation coefficients. “a” represents statistical significance (P < 0.01).

10.5334/jcr.225.s2 Figure S2. **Age-dependent difference in the correlation between M-E preference and time awareness.** The rank correlation analysis in Figure 1 was reperformed by gender. Each blue or red dot indicates data from a male (m) or female (f) subject. ρ values represent correlation coefficients. “a” represents statistical significance (P < 0.01).

10.5334/jcr.225.s3 Supplemental Information. Time Management Scale and Time Anxiety Scale.
