# Supplementary material for: Correlation Analysis Between Time Awareness and Morningness-Eveningness Preference
Source: J Circadian Rhythms. 2023 Oct 11;21:2. doi: 10.5334/jcr.225 (PMC10573578; doi:10.5334/jcr.225)

Figure S1_m/f_fin

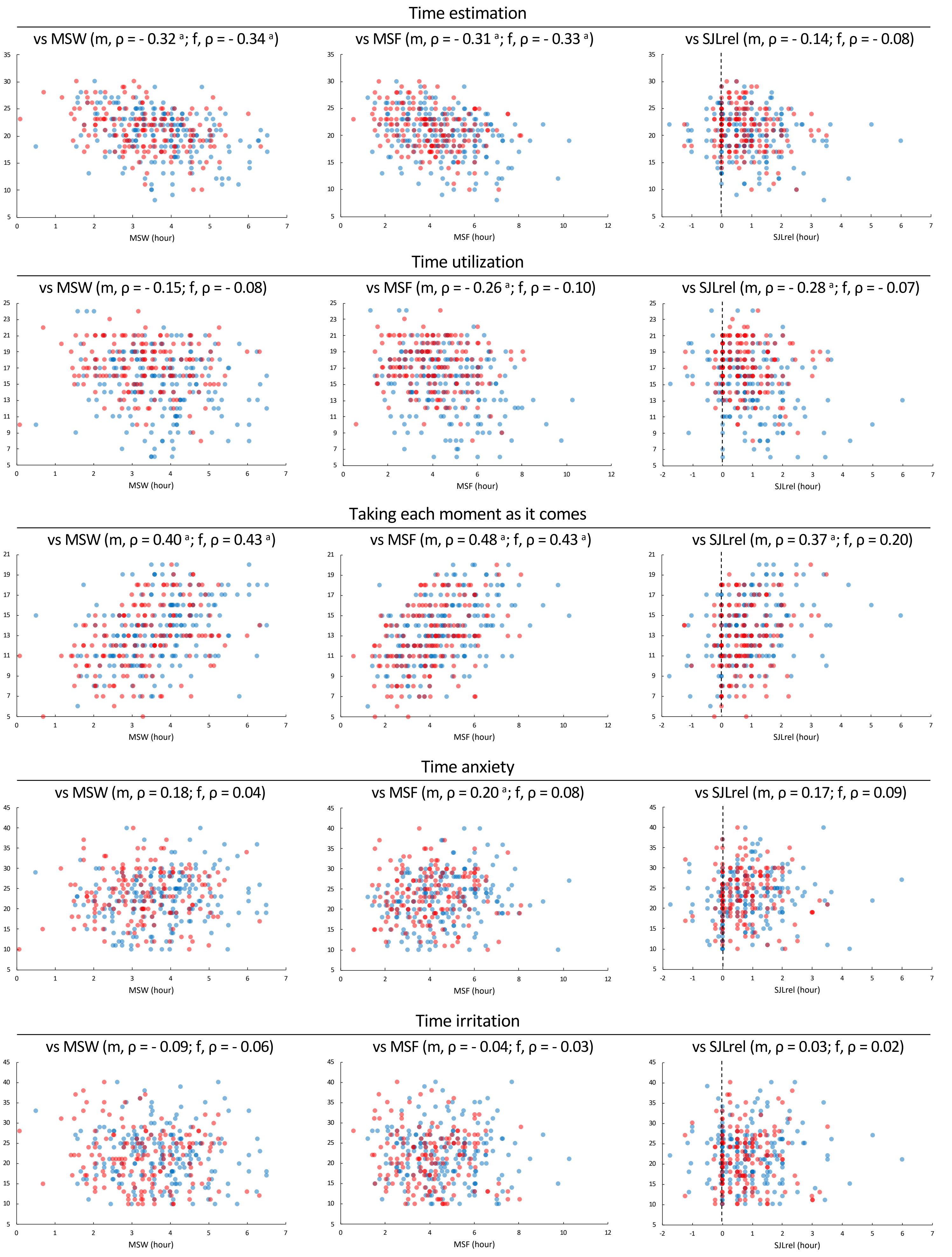

Supplement: Figure S1. — Sex-dependent difference in the correlation between M-E preference and time awareness. The rank correlation analysis in Figure 1 was reperformed by gender. Each blue or red dot indicates data from a male (m) or female (f) subject. ρ values represent correlation coefficients. “a” represents statistical significance (P < 0.01). [file jcr-21-225-s1.pdf]
