# Supplementary material for: Correlation Analysis Between Time Awareness and Morningness-Eveningness Preference
Source: J Circadian Rhythms. 2023 Oct 11;21:2. doi: 10.5334/jcr.225 (PMC10573578; doi:10.5334/jcr.225)

Figure S2_y/o_fin

Time estimation

vs MSW (22 ≥, ρ = - 0.21 [a]; 23 ≤, ρ = - 0.40 [a]) | vs MSF (22 ≥, ρ = - 0.24 [a]; 23 ≤, ρ = - 0.34 [a]) | vs SJLrel (22 ≥, ρ = - 0.15; 23 ≤, ρ = - 0.03)

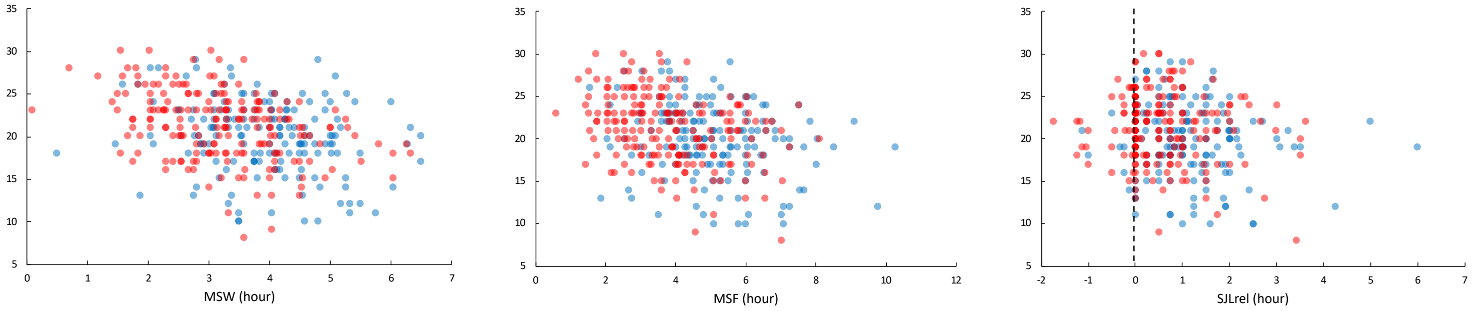

Time utilization

vs MSW (22 ≥, ρ = - 0.04; 23 ≤, ρ = - 0.11) | vs MSF (22 ≥, ρ = - 0.21 [a]; 23 ≤, ρ = - 0.08) | vs SJLrel (22 ≥, ρ = - 0.29 [a]; 23 ≤, ρ = 0.01)

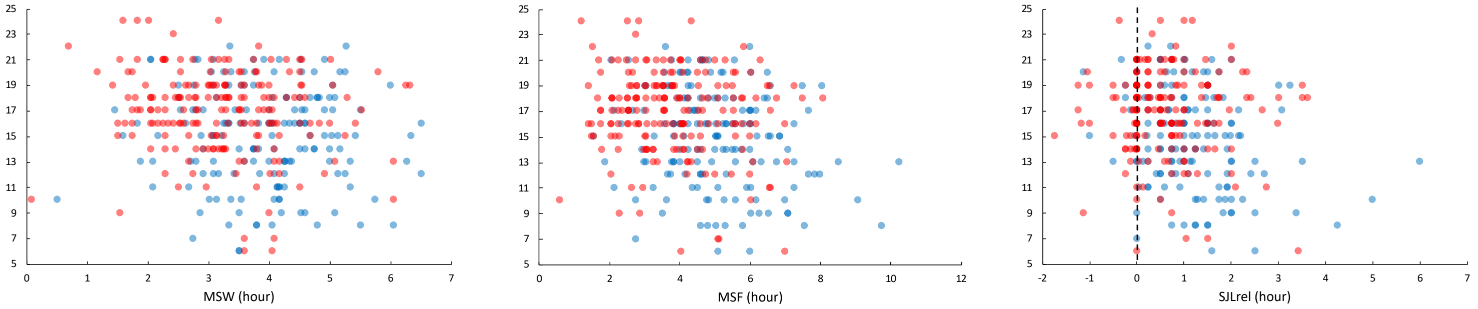

Taking each moment as it comes

vs MSW (22 ≥, ρ = 0.30 [a]; 23 ≤, ρ = 0.40 [a]) | vs MSF (22 ≥, ρ = 0.39 [a]; 23 ≤, ρ = 0.39 [a]) | vs SJLrel (22 ≥, ρ = 0.30 [a]; 23 ≤, ρ = 0.16)

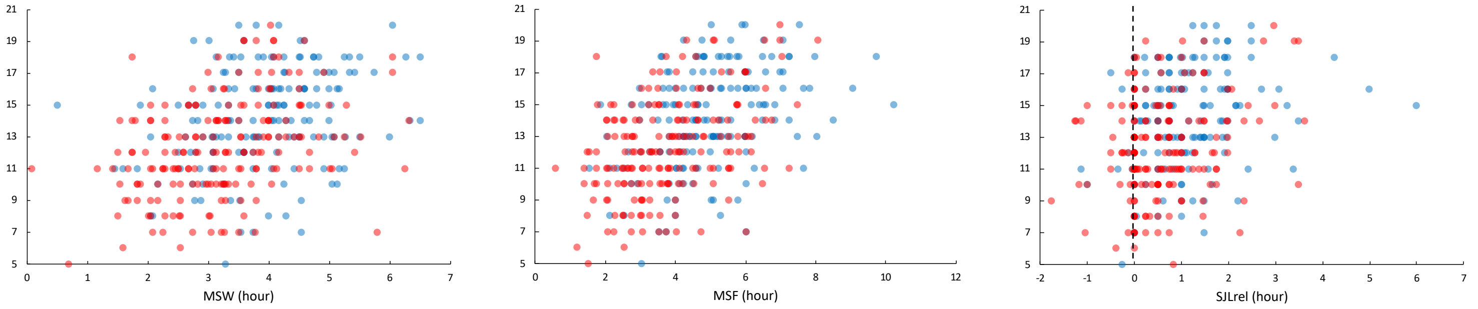

Time anxiety

vs MSW (22 ≥, ρ = 0.14; 23 ≤, ρ = - 0.00) | vs MSF (22 ≥, ρ = 0.16; 23 ≤, ρ = 0.05) | vs SJLrel (22 ≥, ρ = 0.10; 23 ≤, ρ = 0.12)

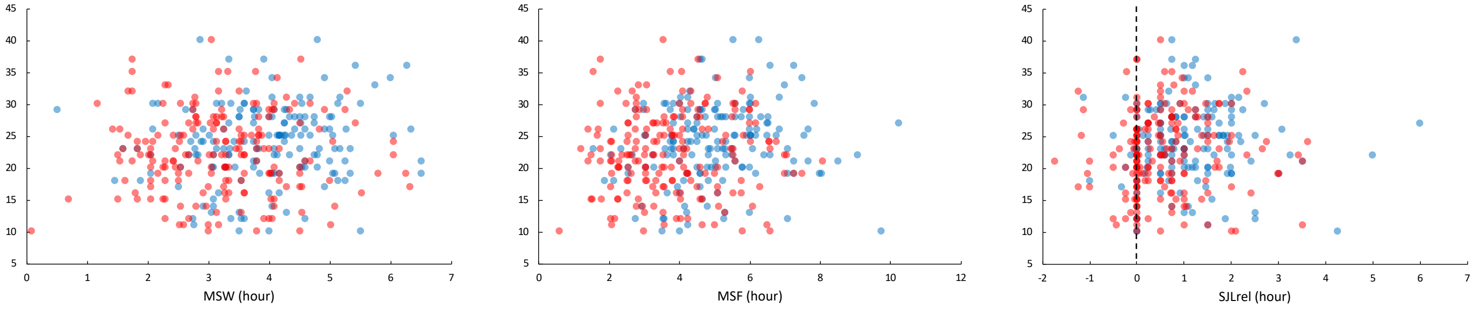

Time irritation

vs MSW (22 ≥, ρ = - 0.03; 23 ≤, ρ = - 0.06) | vs MSF (22 ≥, ρ = 0.07; 23 ≤, ρ = - 0.09) | vs SJLrel (22 ≥, ρ = 0.16; 23 ≤, ρ = - 0.04)

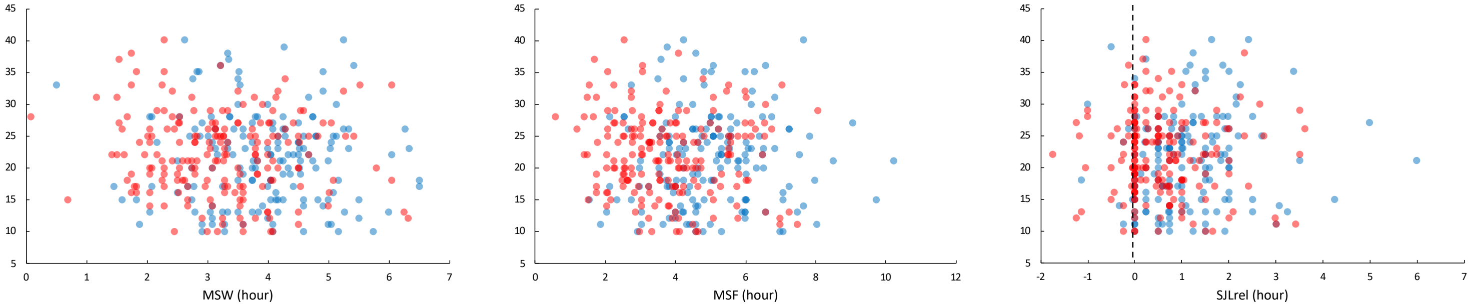

Supplement: Figure S2. — Age-dependent difference in the correlation between M-E preference and time awareness. The rank correlation analysis in Figure 1 was reperformed by gender. Each blue or red dot indicates data from a male (m) or female (f) subject. ρ values represent correlation coefficients. “a” represents statistical significance (P < 0.01). [file jcr-21-225-s2.pdf]
