# Supplementary material for: Correlation Analysis Between Time Awareness and Morningness-Eveningness Preference
Source: J Circadian Rhythms. 2023 Oct 11;21:2. doi: 10.5334/jcr.225 (PMC10573578; doi:10.5334/jcr.225)
Supplement: Supplemental Information. — Time Management Scale and Time Anxiety Scale. [file jcr-21-225-s3.pdf]

Time Management Scale

List of questions:

1. I try to work on my assignments at a set time.
2. I try to set small goals when working on assignments.
3. I try to prepare early for everything so that I have enough time to do it.
4. I try to go to bed and get up early to make as much time as possible available.
5. I estimate in advance how long it will take to do what I want to do.
6. I prioritize the things I have to do over the things I want to do.
7. When I work on a task, I keep track of how much time I spend on it.
8. Once I start something, I don't stop until I finish it.
9. I try to make use of my free time.
10. I make plans for my days off.
11. I use a calendar or a notebook to keep track of my schedule.
12. I check and confirm my schedule for the next day.
13. I prioritize the things I have to do.
14. I keep a diary.
15. I often do not have a plan for the next day.
16. I sometimes act on the spur of the moment or my mood.
17. I always start things at night.
18. I try not to make too many plans.
19. I try not to be lazy on my days off.

Time Anxiety Scale

List of questions:

1. I get confused when things don't go as expected.
2. I get very anxious when I don't get things done.
3. I get upset when I don't have enough time to work on something.
4. I get anxious when I don’t have a plan.
5. I get disturbed when my work is interrupted.
6. I get upset when there is a sudden change of plans.
7. I can't get anything else done until I finish what I'm working on.
8. I can't get started on a task unless I plan for it.
9. I am more pressed for time than others.
10. I don't know what to do when something happens unexpectedly.
11. I get irritated when waiting for a traffic light.
12. I get irritated when waiting in line, even if it's only for a short time.
13. I get impatient with people who are slow to speak.
14. I don't want to waste time waiting for others.
15. I can't tolerate subways or trains that are not on time.
16. I don't want people to disturb my precious time.
17. I feel that the time I spend waiting is too long.
18. I get annoyed when people are late.
19. I feel that it is just a waste of time to spend time waiting.
20. I have no patience for slow workers.
